# Classical theory of universal quantum work distribution in chaotic and disordered non-interacting Fermi systems

**DOI:** 10.1038/s41598-022-18796-3

**Published:** 2022-09-02

**Authors:** András Grabarits, Márton Kormos, Izabella Lovas, Gergely Zaránd

**Affiliations:** 1 Department of Theoretical Physics, Institute of Physics, Budapest University of Technology and Economics, Műegyetem rkp. 3., H-1111 Budapest, Hungary; 2 BME-MTA Exotic Quantum Phases ‘Lendület’ Research Group, Budapest University of Technology and Economics, Műegyetem rkp. 3., H-1111 Budapest, Hungary; 3 MTA-BME Quantum Dynamics and Correlations Research Group, Budapest University of Technology and Economics, Műegyetem rkp. 3., H-1111 Budapest, Hungary; 4 Department of Physics and Institute for Advanced Study, Technical University of Munich, D-85748 Garching, Germany; 5 Munich Center for Quantum Science and Technology (MCQST), Schellingstr. 4, D-80799 München, Germany; 6 Kavli Institute for Theoretical Physics, University of California, Santa Barbara, CA 93106-4030 USA

**Keywords:** Condensed-matter physics, Theoretical physics

## Abstract

We present a universal theory of quantum work statistics in generic disordered non-interacting Fermi systems, displaying a chaotic single-particle spectrum captured by random matrix theory. We consider quantum quenches both within a driven random matrix formalism and in an experimentally accessible microscopic model, describing a two-dimensional disordered quantum dot. By extending Anderson’s orthogonality determinant formula to compute quantum work distribution, we demonstrate that work statistics is non-Gaussian and is characterized by a few dimensionless parameters. At longer times, quantum interference effects become irrelevant and the quantum work distribution is well-described in terms of a purely classical ladder model with a symmetric exclusion process in energy space, while bosonization and mean field methods provide accurate analytical expressions for the work statistics. Our results demonstrate the universality of work distribution in generic chaotic Fermi systems, captured by the analytical predictions of a mean field theory, and can be verified by calorimetric measurements on nanoscale circuits.

## Introduction

One of the fundamental principles of modern physics is emergent universal behavior. In equilibrium, prominent examples of universality are phase transitions, with a critical behavior solely determined by the symmetries and dimensionality of the system, independent of the microscopic details^1,2^. Another striking example of universal behavior is the Fermi liquid theory of interacting fermions, capturing the low temperature properties of most normal metals^3^. In recent years, great effort has been devoted to identify and experimentally observe the universal aspects of out-of-equilibrium dynamics as well^4–6^. Universality has been demonstrated in the non-equilibrium dynamics of near-integrable one-dimensional Bose gases^7,8^, as well as in the form of emergent classical hydrodynamics in the late time relaxation of generic many-body systems^9,10^.

Our main goal in this paper is to extend the powerful paradigm of universality to the rapidly growing field of non-equilibrium quantum thermodynamics. We are motivated by the swift experimental developments in recent years that allow us to study quantum thermodynamics in various systems ranging from individual molecules^11–13^ through mesoscopic grains^14,15^ and nuclear spins^16^ to cold atoms^17^ and nitrogen vacancy centers^18^.

The concepts of heat and work lie at the foundations of thermodynamics; generalizing them to the quantum realm poses new challenges^19–22^. The definition and measurement of work in quantum systems becomes non-trivial, and requires a two-time measurement protocol: one first determines the energy $E^i_{0}$ of the initial state at time $t=0$, and later, in a second measurement, the energy $E^f_{t}$ of the time evolved system at time *t*, with $E^f_{t}$ being an eigenenergy of *H*(*t*). The total energy absorption, $E^f_{t}-E^i_{0}$, can be divided into two contributions. The adiabatic part, denoted by $E^i_{t}-E^i_{0}$, measures the absorbed energy for adiabatically slow processes, where the occupations of instantaneous energy levels do not change during the quantum quench. This contribution incorporates only an essentially trivial time-dependent shift of the energy levels of ${\hat{H}}(t)$. The second, ‘entropic’ contribution of energy absorption or ‘work’, defined as $W \equiv E^f_{t} - E^i_{t}$, accounts for the non-adiabatic particle-hole excitations induced by the quench protocol, i.e., the energy absorbed ($W>0$) or emitted ($W<0$) by the system due to non-adiabatic transitions. In this work, we focus on the non-adiabatic contribution *W*, and investigate the corresponding distribution function, $P_{\,t}(W)$. For simplicity, we consider *quantum quench* protocols, i.e., we start from the ground state of the initial Hamiltonian ${{\hat{H}}}(0)$, but our results can be readily generalized to finite temperature mixed states^23^. The distribution of quantum work is thus given by

1 $$\begin{aligned} P_{\,t}(W)\equiv \Big \langle \delta \big [W-({\hat{H}}(t)-E_\text {GS}(t))\big ]\Big \rangle \,. \end{aligned}$$

  While the full distribution of work in clean many-body systems has been studied quite extensively^20,24–30^ relating it to the Loschmidt echo^24,31^ and to quantum information scrambling^31,32^, in the presence of disorder it was only investigated in the sudden quench limit^33–36^. The effects of disorder were studied for more general protocols, including finite frequency drivings, in the pioneering works^37–43^, however, these focused exclusively on the average energy absorption and did not discuss the full distribution of work.

**Figure 1:**
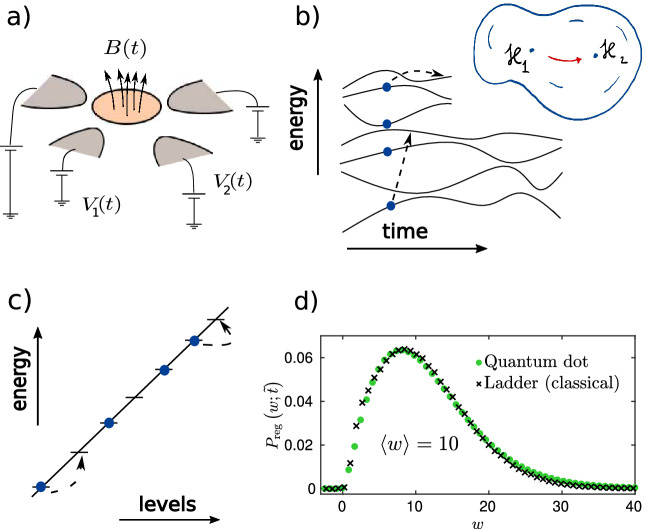
Work statistics in a generic disordered quantum dot. (**a**) Sketch of a quantum dot subject to time dependent magnetic field and gate voltages, realizing a quantum quench and leading to energy absorption. (**b**) Deformation-induced motion of energy levels during the quench giving rise to particle-hole excitations. *Inset:* Quantum quench in a generic disordered dot modelled as a trajectory in the manifold of random matrices. (**c**) ‘Ladder’ model for energy absorption: classical diffusion of hard core particles on uniformly spaced energy levels. (**d**) Benchmarking the work statistics of a realistic quantum dot system against the simplified ladder model for quenches starting from the ground state. Work distribution evaluated from the full microscopic description of the quantum dot, and the results within the classical ladder model collapse onto a universal curve. The values of the numerical parameters are the same as in Fig. 3 below.

To fill this gap, and to examine the universal aspects of the work statistics of Fermi liquids, we focus on disordered, chaotic fermion systems such as 2-dimensional quantum dots, which we perturb by changing external gate voltages, fields, and electrodes, as shown in Fig. 1a. We neglect interactions under the assumptions that a non-interacting Fermi liquid description is appropriate. We note that these systems are chaotic in the sense that their level statistics is well captured by random matrix theory, however, due to the lack of interactions, they are not ergodic in the many-body sense. Under these conditions, the system can be described in terms of the time dependent Hamiltonian

2 $$\begin{aligned} \hat{H}(t)=\sum _{i,j=1}^N \hat{a}_i^\dagger \,\mathscr {H}_{ij}(t)\,\hat{a}_j\,, \end{aligned}$$

where the $\hat{a}_i$’s stand for fermionic annihilation operators, and the single particle Hamiltonian $\mathscr {H}(t)$ incorporates disorder effects and also accounts for the impact of time dependent electrodes. The total fermion number is conserved by Eq. (2), $\sum _i \hat{a}_i^\dagger \hat{a}_i = M$.

We study the universal features of the work statistics for zero temperature quench protocols, starting from the ground state of the initial Hamiltonian, in two steps. First, relying on the observation that the single particle spectrum of most chaotic systems is captured by random matrix theory (RMT)^44–46^, we investigate quenches within random matrix ensembles, such that $\mathscr {H}(t)$ follows a trajectory within the manifold of Gaussian random matrices (see the inset of Fig. 1b). Secondly, after establishing the universal properties of work distribution within this random matrix description, we turn to a more realistic microscopic model of a disordered Fermi liquid. We ask the natural question whether the universal features predicted by random matrix theory remain valid for a more realistic, experimentally accessible disordered tight binding Hamiltonian. We find that the answer to this question is affirmative; our results demonstrate the striking universality of the work statistics both within a random matrix framework and for a full microscopic description (see Fig. 1d).

To set up the framework of random matrix theory, we follow the strategy of Refs.^39,47^ and^48^, and consider deformations within the space of Gaussian random matrix ensembles,

3 $$\begin{aligned} \mathscr {H}(t)=\mathscr {H}_1\cos \lambda (t)+\mathscr {H}_2\sin \lambda (t)\;, \end{aligned}$$

 with $\mathscr {H}_{1,2}$ some independent $N\times N$ Gaussian matrices from the orthogonal (GOE), unitary (GUE) or symplectic (GSE) ensembles, inducing the motion and collisions of single-particle energy levels, see Fig. 1b. Here ${\dot{\lambda }} = v$ sets the speed of deformations and generates a motion along an ’arc’ or ’circle’ within the random matrix ensemble, as depicted in the inset of Fig. 1b. Our first goal is to understand universal aspects of the structure and time evolution of the distribution $P_t(W)$ within this framework, with the initial state chosen as the ground state of ${{\hat{H}}}(0)$. We follow the quantum evolution of the disordered many-body systems, and use a determinant formula presented in Ref.^48^ to compute $P_t(W)$. Our first main finding is that the statistics of $P_t(W)$ is almost independent of microscopic details as well as the symmetry of the Hamiltonian, once the absorbed energy exceeds sufficiently the one-body energy separation $\delta \varepsilon \equiv 1/N(\varepsilon _F)$, characterizing the total density of levels at the Fermi energy $\varepsilon _F$, and the time is long enough, $t>\hbar /\delta \varepsilon$. To capture work in this long time limit, we construct a simplified *classical* ‘ladder’ model which only incorporates Pauli exclusion and level repulsion to the lowest order, in the form of a completely rigid spectrum. However, the ‘ladder’ model ignores all additional features of the level statistics, including its dependence on the random matrix ensemble parameter $\beta$, as well as the interference effects between consecutive level collisions and Landau–Zener transitions, see Fig. 1c. Our simplified ‘ladder’ model gives a surprisingly accurate description of $P_t(W)$, underlining the high level of universality in the work statistics, and allowing us to derive accurate *analytical* approximations for $P_t(W)$ by means of bosonization and a particle number conserving mean field method. These analytical formulas constitute the second main result of our work. We then turn to our last main objective, and provide further evidence for universality of the work statistics by going beyond the RMT framework. By comparing the ‘ladder’ model to a realistic 2D quantum dot system, we confirm the striking universality of the work statistics for the full microscopic description, thereby validating our random matrix theory approach (see Fig. 1d).

## Results

### Quantum mechanical analysis

In this section we briefly summarize the main steps allowing us to numerically evaluate the full distribution of work, $P_t(W)$, for an arbitrary quadratic Hamiltonian, Eq. (2), following Ref.^48^. For a non-interacting Hamiltonian, all information is contained in the time evolution of the single particle wave functions, ${\varvec{\varphi }}^m(t)$, that evolve according to the Schrödinger equation ($\hbar =1$)

4 $$\begin{aligned} i\,\partial _t{\varvec{\varphi }}^m(t) ={{\mathscr {H}}}(t) {\varvec{\varphi }}^m(t) \end{aligned}$$

with initial conditions $\varphi _i^m(0)= \delta ^m_i$. We solve Eq. (4) using the adiabatic approach, by expanding ${\varvec{\varphi }}^m(t)$ in terms of the instantaneous eigenfunctions ${\varvec{\eta }}^k_t$ of ${\mathscr {H}}$ satisfying ${{\mathscr {H}}}(t)\, {\varvec{\eta }}_{\,t}^m= \varepsilon _m(t) {\varvec{\eta }}_{\,t}^m$ as

$$\begin{aligned} {\varvec{\varphi }}^m(t) =\sum _k\alpha ^m_k(t) \,{\varvec{\eta }}^k_t\,. \end{aligned}$$

We then solve the single particle Schrödinger equation for $\alpha ^m_k(t)$ with initial conditions $\alpha _k^m(0)=\delta _k^m$,

5 $$\begin{aligned} i \,{\dot{\alpha }}_k(t)= \varepsilon _k(t) \alpha _k(t) + \sum _{l} A^{kl}(t)\,\alpha _l(t)\,, \end{aligned}$$

where $A^{kl} = -i \; {\varvec{\eta }}^k_t \cdot \partial _t {\varvec{\eta }}^l_t$ is the Berry connection. For zero temperature quenches, the characteristic function $G_t(u)$ of the work distribution $P_t(W)$ can then be expressed by a simple determinant formula^48,49^

6 $$\begin{aligned} G_t(u)= \big \langle \big \langle \Psi (t)|\, e^{iu\left( \hat{H}(t)-E_\text {GS}(t)\right) }\,|\Psi (t)\big \rangle \big \rangle _\text {disord} =\big \langle e^{-i\,u\sum _{m=1}^{M}\varepsilon _m(t)}\,\mathrm{det}\, g_t(u)\big \rangle _\text {disord}\;. \end{aligned}$$

Here $|\Psi (t)\rangle$ is the full time-evolved many-body wave function, with $|\Psi (0\rangle$ being the ground state of the initial Hamiltonian ${\hat{H}}(0),$ $E_\text {GS}(t)$ is the ground state energy of the instantaneous Hamiltonian ${\hat{H}}(t)$, and $\langle \dots \rangle _{\mathrm {disord}}$ denotes the averaging over disorder. The matrix $g_t(u)$ contains information on overlaps and the instantaneous single particle energies $\varepsilon _k(t)$ at time *t*,

7 $$\begin{aligned} \left[ g_t(u)\right] ^{m m^\prime }\equiv \sum _k [\alpha _k^m(t)]^*\,e^{i\,u\,\varepsilon _k(t)}\,\alpha _k^{m^\prime }(t)\,. \end{aligned}$$

We compute $g_t(u)$ numerically, average over disorder or the random matrix ensemble, $\langle \dots \rangle _{\mathrm {disord}}$, and determine the final distribution by performing a Fourier transformation.

The average level spacing $\delta \varepsilon$ and its inverse provide natural energy and time scales, and allow us to introduce the dimensionless work and time, $w\equiv W/\delta \varepsilon$ and $\tilde{t} \equiv t\,\delta \varepsilon$, respectively. As shown in Fig. 1b, deformations of the Hamiltonian lead to a continuous motion of single particle levels, and thereby induce collisions and transitions between them. At longer times, these collisions and Landau–Zener transitions^38,40,47,50^ give rise to a *diffusive* broadening of the Fermi surface, more precisely, of the Fermi edge in energy space, since momentum is not well-defined in the presence of disorder. Namely, the mean occupation of the *k*th energy level is given by $f_k\left( {t}\right) =\left( {1-t_k\left( {t}\right) }\right) /2$ with $t_k(t)=\mathrm {erf}\left( \Delta k/\sqrt{4{\widetilde{D}}{\tilde{t}}}\right)$ where $\Delta k = k-M-1/2$ is measured from the Fermi-level, and ${\widetilde{D}}$ is the dimensionless energy diffusion constant.

After a short time perturbative $\sim t^2$ scaling, the average work is found to increase as $\langle w\rangle = {\widetilde{D}} \,{\tilde{t}}$ (see Refs.^37,38,40,48^ and Methods). Let us note that quantum effects such as dynamical localization yielding time dependent corrections to the diffusion constant studied for periodic driving in the important early works^41–43^ become only relevant on a time scale much larger than the period^39^ and are thus negligible on our time scales. Below we turn to the full distribution of the dimensionless work *w* in different settings, both numerically and through various analytical approximations.

### Quenches in random matrix ensembles

In this section we analyze the works statistics within the framework of random matrix theory for quenches of the form of Eq. (3). The disorder average appearing in Eq. (6) becomes an average over two independent random matrices, $\mathscr {H}_{1,2}$. The distribution $P_{\,\tilde{t}\,}(w)$ can be disentangled into an adiabatic and a regular part,

8 $$\begin{aligned} P_{\,{\tilde{t}}\,}(w)=P_\text {ad}(\,{\tilde{t}}\,)\, \delta (w)+P_\mathrm{reg}(w;\,{\tilde{t}}\,)\;. \end{aligned}$$

Random matrix theory implies that - apart from the symmetry of the Hamiltonian - the statistics of the evolution of the eigenvalues, sketched in Fig. 1b, is completely characterized by the *velocity* with which levels deform, i.e., the frequency of avoided level crossings. Indeed, the average distance of level crossings^47,50,51^, $\langle \Delta \lambda \rangle$, and the time scale $1/\delta \varepsilon$ define a natural ‘velocity’ in parameter space, $v_c \equiv \langle \Delta \lambda \rangle \delta \varepsilon$, which we can use to introduce the dimensionless velocity, ${\tilde{v}} \equiv {{\dot{\lambda }}} / ( \langle \Delta \lambda \rangle \delta \varepsilon )$. For $N\times N$ random matrices, $\delta \varepsilon \sim 1/N$, and $\langle \Delta \lambda \rangle \sim 1/\sqrt{N}$, therefore $v_c\sim 1/N^{3/2}$. The dimensionless velocity characterizes microscopic processes. For ${\tilde{v}} \ll 1$ the motion is almost adiabatic, and small probability Landau–Zener transitions dominate. For $\tilde{v} \gg 1$, on the other hand, transitions between remote levels generate energy absorption.

From our random matrix considerations it follows that the distribution $P_{\,{\tilde{t}}}(w)$ can only depend on ${\tilde{t}}$, ${\tilde{v}}$, and, in case of finite temperature initial states, on the dimensionless initial temperature, ${\widetilde{T}} \equiv T/\delta \varepsilon$. Similarly, the diffusion constant ${\widetilde{D}}$ is a universal function of ${\tilde{v}}$, which scales as ${\widetilde{D}}\sim {\tilde{v}} ^2$ for large velocities, while for ${\tilde{v}} <1$ nearest neighbor transitions dominate and yield ${\widetilde{D}}\sim {\tilde{v}} ^{(\beta /2 +1)}$, with $\beta = 1,2$ and 4 characterizing the orthogonal, unitary, and symplectic ensembles, respectively (see the Methods for more details).

**Figure 2:**
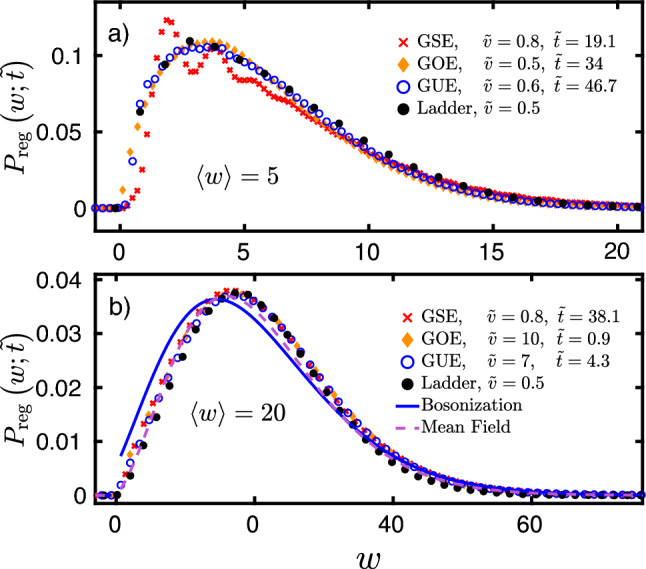
Work statistics for GSE, GOE, GUE, for dimensionless average work $\langle w\rangle =5$ (**a**), and $\langle w\rangle =20$ (**b**). For smaller $\langle w\rangle$, $P_\mathrm{reg}(w;\,{\tilde{t}}\,)$ displays features associated with level repulsion and specific to the symmetry of the underlying Hamiltonian, while for large $\langle w\rangle$, the distributions $P_{\mathrm{reg}}(w;\,{\tilde{t}}\,)$ fall onto a single universal curve for all RMT universality classes and velocities, captured by a classical ladder model. Mean field (dashed line) and bosonization (continuous line) approaches give accurate description in the diffusive regime. In panel a), the number of energy levels was $N=28$ for the GSE, $N=20$ for the GOE and GUE, and $N=80$ for the ladder model simulations. In panel b), these numbers are $N=96$ (GSE), $N=50$ (GUE), $N=52$ (GOE), and $N=70$ (ladder model). The number of particles was set to half filling, $M=N/2$.

We show our random matrix simulation results in Fig. 2. We checked that the work distribution is not sensitive to changes in the number of energy levels *N* and the number of particles *M*, as long as the particle-hole excitations contributing to the work are created far from the edges of the band (see also Ref.^48^.) For small work, $\langle w\rangle \lesssim 10$, the statistics depends on $\beta$ as well as on ${\tilde{v}},$ and $P_{\mathrm{reg}}(w;\,{\tilde{t}}\,)$ displays peaks and minima associated with level repulsion, clearly reflecting the symmetry of the underlying Hamiltonian (see Fig. 2a). These features become more pronounced for larger $\beta$ due to the stronger level repulsion. For larger works, $\langle w\rangle \gtrsim \max \, \{{\tilde{v}}^2,1\}$, however, one enters a diffusion dominated regime, where symmetry related and microscopic features become less important, and a universal distribution displayed in Fig. 2b emerges. The observed distribution is clearly non-Gaussian, and characterizes work statistics in generic non-interacting fermion systems.

### Ladder model

**Figure 3:**
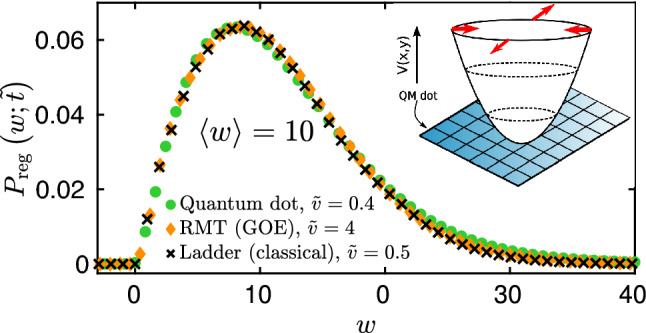
Work statistics for dimensionless average work $\langle w\rangle =10$. Microscopic quantum dot model (*inset*) simulations (green circles), random matrix (GOE) results (orange diamonds), and the ‘ladder’ model statistics (black crosses) fall on top of each other with good accuracy. Quantum dot calculations were performed for $M=427$ electrons for a lattice of size $38\times 38$, disorder variance $\sigma =1.75J$, potential strength $\alpha =75J$, deformation amplitude $\lambda _\text {f}=0.1,$ and dimensionless velocity ${\tilde{v}}=0.4$. For the GOE computations we used $N = 40$ with $M=20$ electrons, and velocity ${\tilde{v}}=4$. For the ‘ladder’ model simulations we used ${\tilde{v}}=0.5,$ and $N=120$ levels with $M=60$ electrons. Quantum work distribution depends only on the average work $\langle w\rangle$ and is well captured by the classical ‘ladder’ model.

The agreement between the three universality classes is suggestive that quantum interference effects do not play an important role in this diffusion-dominated regime. We can therefore attempt and construct a classical *‘ladder’* model, consisting of uniformly placed classical energy levels at a distance $\delta \varepsilon$ from each other (see Fig. 1c),

9 $$\begin{aligned} \varepsilon _k = k\,\delta \varepsilon \,,\quad \quad k=1,2,\dots \,, \end{aligned}$$

occupied by hard-core particles in line with Fermi statistics. The energy of a many-body state is then given by $E=\sum _k n_k\,\varepsilon _k$ with $n_k \in \{0,1\}$ the occupation numbers, and $\sum _k n_k = M$ the total number of particles. The evenly placed levels (9) mimic level repulsion and level rigidity in chaotic systems. As a final component, perturbation-induced random Landau–Zener transitions are modeled by nearest neighbor hopping transitions and a symmetrical exclusion process (SEP) in energy space. The SEP is a classical Markovian stochastic system of hard-core particles trying to jump at a constant rate to the left or to the right with probability 1/2 along a 1D lattice with the constraint that no site can be occupied by more than one particle (exclusion property). This simple model captures the diffusive broadening of the Fermi surface (see Methods) and, in addition to level repulsion, it also incorporates Fermi statistics and particle number conservation. In our SEP simulations, the jump rate was set by the diffusion constant ${\widetilde{D}}$ such that for a given quench time the same final occupation profile and average work is obtained as in the quantum simulation. As can be seen in Figs. 2 and 3, this classical stochastic model gives a surprisingly accurate description of the work statistics for large enough average work, independently of the velocity. Moreover, with certain assumptions, the ‘ladder’ model can be used to compute $P_\mathrm {\,ad}(\,{\tilde{t}}\,)$ and $P_{\mathrm{reg}}(w;\,{\tilde{t}}\,)$ analytically for a $T=0$ temperature initial state, without performing the actual Monte Carlo simulations, using either bosonization or a more accurate mean field approach. It is, however, crucial to treat particle number conservation with care.

### Bosonization

Bosonization offers a simple method to treat particle number conservation in the ‘ladder’ model. Introducing fermion operators for each level, we can express the total energy as $H=\sum _k (\varepsilon _k-\varepsilon _F) :c^\dagger _k c_k:$

  with $\varepsilon _F = \delta \varepsilon \,(M+1/2)$ the Fermi energy and  : ... :  referring to normal ordering with respect to the Fermi sea. Following Ref.^52^, we introduce bosonic operators, $b^\dagger _{q>0}\equiv (1/\sqrt{q})\, \sum _k c^\dagger _{k+q}c_k$, which satisfy the usual commutation relations, $[b_q,b^\dagger _{q^\prime }] = \delta _{q,q^\prime }$, and rewrite the Hamiltonian in terms of these as

10 $$\begin{aligned} H = \sum _{q\in \mathbb {Z}^+} \delta \varepsilon \, q \; b^\dagger _q b_q + \frac{\delta \varepsilon }{2} { {\hat{N}}}^2 \end{aligned}$$

 with ${\hat{N}} = \sum _k c^\dagger _k c_k -M$ the normal ordered fermion number. Clearly, the fermion number does not change for the closed system studied here so the second term in Eq. (10) does not give a contribution. We can obtain an approximate expression for $P_{\,{\tilde{t}}}(w)$ by assuming that the final state is thermal with an effective boson temperature ${{\widetilde{T}}}_\mathrm {eff} = \sqrt{6\langle w\rangle } / \pi$, chosen to yield the appropriate average energy, $\langle \sum _{q>0} q \; b^\dagger _q b_q \rangle \equiv \langle w\rangle$. In the large $\langle w\rangle$ limit, we then obtain (for details see Methods),

11 $$\begin{aligned} P^\mathrm {\,Bose}_{{\tilde{t}}}( w) \approx e^{-\frac{\pi ^2 {\widetilde{T}}_\mathrm {eff}}{6}} \, \Bigl [\frac{\pi }{\sqrt{6\, w}}e^{-w/{\widetilde{T}}_\text {eff}} \;{I}_1\bigl (\pi \sqrt{\textstyle {\frac{2}{3}}w}\bigr ) +\delta \left( w\right) \Bigr ], \end{aligned}$$

where $I_1$ is the modified Bessel function of the first kind. Since $T_\mathrm {eff}\sim \sqrt{{\widetilde{D}} {\tilde{t}}}$, the prefactor decays as $\sim e^{- C \sqrt{{\widetilde{D}} {\tilde{t}}}}$, corresponding to a stretched exponential decay of adiabatic processes, as confirmed by our quantum mechanical simulations^48^.

### Mean field theory

The bosonization approach yields a good account of the overall structure of $P_{{\tilde{t}}}(w)$, but with certain limitations (see Fig. 2b). In particular, the assumption of a thermal final state is not quite correct. The occupation of the single particle levels after the time evolution is not described by the Fermi function but has a diffusive structure, as stated earlier. A more accurate expression can be obtained for $P_{\,{\tilde{t}}}(w)$ in a simple, particle number conserving *mean field* approach, where instead of assuming thermalization, we rely on the diffusive nature of energy absorption, and assume that each fermion level *k* is occupied with probability $f_k = (1-\mathrm {erf}\left( \Delta k/\sqrt{4{\widetilde{D}}{\tilde{t}}}\right) )/2$ with $\Delta k = k-M-1/2$ measured from the Fermi-level, corresponding to a diffusive broadening of the Fermi surface. To enforce the constraint, $\sum _k n_k = M$, we use an integral representation over an auxiliary variable. A saddle point procedure in this latter then yields accurate expressions for $P_\text {\,ad}(\,{\tilde{t}}\,)$ as well as for $P_{\mathrm{reg}} (w;\,{\tilde{t}}\,)$.

The mean field probability distribution, $P^{\mathrm{MF}}_{\,{\tilde{t}}} (w)$, is similar in structure to Eq. (11), but contains additional correction terms (see Methods for details),

12 $$\begin{aligned} P^\mathrm {\,MF} _{{\tilde{t}}}(w) \approx P_{\,\mathrm {ad}}^\mathrm {\,MF}\; \delta (w)\, + \frac{c_w}{\sqrt{w}} \, e^{-c_w \frac{w + {\langle w\rangle }}{\sqrt{{\langle w\rangle }}}} \left[ I_1 (2c_w\sqrt{w}) - \sqrt{2} \,I_1 \bigl ( 2c_w\sqrt{ w/2}\bigr )\right] \end{aligned}$$

with $$c_w\approx 1.35$$ and

13 $$\begin{aligned} P^\mathrm {\,MF}_\text {\,ad} = (8\pi \langle w \rangle )^{1/4}\,{e^{-c_w\sqrt{\langle w \rangle }}}\,. \end{aligned}$$

As shown in Fig. 2b, the mean field expressions above yield an accurate description of work in the diffusive regime. Similar to the bosonization result, Eq. (11), $P^{\mathrm{MF}}_{\,{\tilde{t}}} (w)$ is non-Gaussian and, by construction, depends parametrically only on $\langle w\rangle$. The probability of adiabatic processes also falls off as a stretched exponential, but the prefactor $c_w$ is more accurate than the one obtained by the simple bosonization theory ($\pi /\sqrt{6}\approx 1.28$) (see Ref.^48^ and Methods).

### Validation by microscopic models and experimental setup

The universal features of the work statistics discussed so far have been established within the framework of random matrix theory. It is a natural question to that extent do these result remain valid outside this framework, in realistic disordered systems. To answer this question and to confirm our predictions as well as to validate the results of our random matrix approach, we propose to study a 2-dimensional quantum dot, and squeeze the electron gas confined there by applying time dependent external gate voltages (see Fig. 1a). This system can be realized experimentally^53,54^.

We model the quantum dot by a disordered tight binding Hamiltonian on a square lattice,

14 $$\begin{aligned} {\hat{H}} = - J \sum _{{\mathbf {r}},\varvec{\delta }} {\hat{c}}^\dagger _{{\mathbf {r}}+\varvec{\delta }} \,{\hat{c}}_{{\mathbf {r}}} + \sum _{\mathbf {r}}(V({\mathbf {r}},t) + \varepsilon _{\mathbf {r}}) {\hat{c}}^\dagger _{\mathbf {r}}{\hat{c}}_{\mathbf {r}}\;, \end{aligned}$$

where the sum over $\varvec{\delta }$ runs over the two orthogonal lattice vectors. The first term accounts for the kinetic energy of the electrons, while $\varepsilon _{\mathbf {r}}$ are random onsite energies drawn from a Gaussian distribution of variance $\sigma$, and are responsible for electron scattering and disorder. We emphasize that we focus on the delocalized regime of the model, where the Anderson localization length is much larger than the system size. The potential

15 $$\begin{aligned} V({\mathbf {r}},t) = \frac{1}{2} ( \alpha r^2 + \lambda (t) (x^2-y^2) ) \end{aligned}$$

describes the parabolic confinement, generated by external gate electrodes. The second term in $V({\mathbf {r}},t)$ describes a compression (decompression) of the electron gas in the *x* direction with a simultaneous decompression (compression) along the *y* direction. We vary $\lambda$ linearly in time between $-\lambda _\text {f}$ and $\lambda _\text {f}$ to induce deformations and generate dissipation.

A numerical investigation of the single particle spectrum of Eq. (14) reveals that, although some deviations are clearly present, the spectrum of Eq. (14) is reasonably described in terms of GOE for each value of $\lambda$ (see Methods).

Similarly to the RMT simulations, we generate work by varying lambda at a constant pace, ${{\dot{\lambda }}}=v,$ corresponding to the velocity of potential deformations, and compute work statistics $P_{\tilde{t}}(w)$ according to the formula used for the random matrix models, Eq. (6). In order to get universal work distribution, we introduce the same velocity and time units, however, their dependence on the number of lattice sites, on-site disorder strength, and potential strength is quite different from that in the RMT model. In particular, both $\delta \varepsilon$ and the typical distance between avoided crossings strongly depend both on the potential strength, system size, on-site disorder strength, and the position in the energy spectrum which we, therefore, computed numerically, averaging over $\sim 5\times 10^3$ disorder realizations. Then setting the average work in these units to $\langle w\rangle =10,$ we computed the regular part of the work statistics, $P_\mathrm {reg}(w;{\tilde{t}})$, for velocity ${\tilde{v}}=0.4$, presented in Fig. 3.

The results show striking agreement with random matrix theory as well as with the ‘ladder’ model, and thereby provide further evidence for universality.

An alternative experimental platform to study quantum work statistics is offered by ultracold atoms^17^. For a forward-backward protocol $P_\mathrm {ad}$ is essentially the ground state fidelity, which has been measured in Ref.^55^ by preparing two identical copies of a quantum system, and measuring their overlap. This method could be used to verify the predicted stretched exponential behavior of $P_\mathrm {ad}$ in disordered fermion systems.

## Discussion

We demonstrated universal behavior in the quantum thermodynamics of a chaotic Fermi liquid, displaying a level statistics captured by random matrix theory, by studying the work statistics for quantum quenches in disordered non-interacting fermionic systems. We note that according to Fermi liquid theory, interaction effects are expected to remain irrelevant at single-particle energies $\varepsilon$ such that $(\varepsilon -\varepsilon _F)^2/\varepsilon _\text {F}$ is smaller than the typical level spacing.

First we focused on quenches formulated within the framework of random matrix theory. Strikingly, we found that for large enough average work, the distribution is independent of the random matrix ensemble and is very well captured by a universal *classical* stochastic model describing diffusion in energy space. This simplified ‘ladder’ model only relies on Pauli exclusion principle and on the rigidity of the random matrix spectrum, but neglects all further microscopic details, including the symmetry class of the Hamiltonian. This high level of simplification allowed us to derive approximate analytical expressions both via bosonization and mean field theory. Interestingly, the bosonization result in Eq. (11) also emerged in the context of work statistics in Luttinger liquids after an interaction quench^26^. While the bosonization approach performs more poorly in comparison with the mean field treatment (cf. Fig. 2b), it relies on the additional approximation that the final state is thermal. In contrast, the mean field approach incorporates the more accurate diffusive occupation profile of the final state, and yields a striking agreement with the results of full quantum simulations, thereby providing an excellent analytical approximation for the universal work statistics. Finally, we turned to a more realistic, experimentally accessible microscopic model, and studied the work distribution of a 2D disordered quantum dot subject to time-dependent gate voltages. We demonstrated the striking agreement between the work statistics of this microscopic model and our random matrix theory predictions. These results show that our universal description holds beyond the framework of random matrix theory, and accurately captures the energy absorption in realistic models. Our results could be tested experimentally by studying squeezed disordered quantum dots.

## Methods

## Bosonization approach

In this approach,

we consider an equilibrium fermionic system with uniformly spaced one-particle energy levels. In the framework of bosonization, the fermionic particle-hole excitations with respect to the ground state are represented as bosonic states. We assign thermal Boltzmann weights $e^{-\beta _\mathrm {eff}\,q\delta \varepsilon }$ to these states, where $\beta _\mathrm {eff}$ is an effective inverse temperature while $q\,\delta \varepsilon$ with $q=1,2,\dots$ measures the energy of the particle-hole excitation.

Since these excitations are bosonic, for each *q* we can have $n_q=0,1,2,\dots$ arbitrarily many bosonic excitations with energy $qn_q\delta \varepsilon$. In the characteristic function each of them carries a contribution of $e^{i{\tilde{u}} qn_q}$, so we have

16 $$\begin{aligned} \begin{aligned} G_{{\tilde{t}}}^\text {Bose}\left( {u}\right)&=\mathscr {N}^{-1}\sum _{n_1,n_2,\dots }e^{-\left( {\beta _\mathrm {eff}\delta \varepsilon -i\tilde{u}}\right) n_1}e^{- \left( {\beta _\mathrm {eff}\delta \varepsilon -i \tilde{u}}\right) 2n_2}e^{-\left( {\beta _\mathrm {eff}\delta \varepsilon -i\tilde{u}}\right) 3n_3}\dots =\mathscr {N}^{-1} \prod _{q=1}^\infty \sum _{n_q=0}^\infty e^{-qn_q\left( {\beta _\mathrm {eff}\delta \varepsilon -i{\tilde{u}}}\right) }\\&=\mathscr {N}^{-1}\prod _{q>0}\frac{1}{1-e^{-q\left( {\beta _\mathrm {eff}\delta \varepsilon -i {\tilde{u}}}\right) }}, \end{aligned} \end{aligned}$$

where $\mathscr {N}=\prod _{q>0}\left[ 1-e^{-q\left( {\beta _\mathrm {eff}\delta \varepsilon }\right) }\right] ^{-1}$ so that $G_\mathrm {eff}(0,T_\mathrm {eff})=1$. Exponentiating Eq. (16) and taking the continuum limit $\sum _{q>0}\rightarrow \int _0^\infty \mathrm dx$ we get:

17 $$\begin{aligned} G_{{\tilde{t}}}^\text {Bose}\left( {u}\right) \approx \mathscr {N}^{-1}e^{-\int _0^\infty \mathrm dx\ln \left[ {1-e^{-\left( {\beta _\mathrm {eff}-iu}\right) x}}\right] }=e^{\frac{\pi ^2/6}{\beta _\mathrm {eff}-i\tilde{u}}-\frac{\pi ^2/6}{\beta _\mathrm {eff}}}\,. \end{aligned}$$

The Fourier transform can be performed exactly,

18 $$\begin{aligned} \begin{aligned} \int _{-\infty }^\infty \frac{\mathrm du}{2\pi }e^{-iuw}e^{\frac{\pi ^2/6}{\beta _\mathrm {eff}-iu}}&=\sum _{n=0}^ \infty \frac{\left( {\pi ^2/6}\right) ^n}{n!}\int _{-\infty }^\infty \frac{\mathrm du}{2\pi }\frac{e^{-iuw}}{\left( {\beta _ \mathrm {eff}-iu}\right) ^n}=e^{-\beta _\mathrm {eff}w}\sum _{n=1}^\infty \frac{\left( {\pi ^2/6}\right) ^nw^{n-1}}{n!\left( {n-1}\right) !}+\delta \left( {w}\right) \\&=\frac{\pi }{\sqrt{6w}}e^{-\beta _\mathrm {eff}w}{I}_1\bigl (\pi \sqrt{\textstyle {\frac{2}{3}}w}\bigr )+ \delta \left( {w}\right) , \end{aligned} \end{aligned}$$

which together with the normalisation factor leads to the analytical result expressed in terms of the dimensionless effective temperature

19 $$\begin{aligned} P^\mathrm {\,Bose}_{{\tilde{t}}}( w) \approx e^{-\frac{\pi ^2 {\widetilde{T}}_\mathrm {eff}}{6}} \, \Bigl [\frac{\pi }{\sqrt{6\, w}}e^{-w/{\widetilde{T}}_\text {eff}} \;{I}_1\bigl (\pi \sqrt{\textstyle {\frac{2}{3}}w}\bigr ) +\delta \left( w\right) \Bigr ]. \end{aligned}$$

### Mean field approach

In this section we provide some details about the mean field theory calculations and the resulting analytic expressions.

#### Probability of adiabaticity

Within the mean field approach, the probability of each many-body configuration takes the form of the product of independent Bernoulli weights of *M* occupied and $N-M$ empty sites. In order to simplify calculations and without any loss of generality we consider the case of $M=N/2$:

20 $$\begin{aligned} P\left( \{n_k\}\right) =\dfrac{1}{\mathscr {N}_t}\prod _{k=1}^N p_{k,t}(n_k)\; \delta _{N/2=\sum _k n_k} =\dfrac{1}{\mathscr {N}_t}\int _{-\pi }^{\pi }\dfrac{\mathrm{d}\lambda }{2\pi }e^{i\lambda \sum _{k=1}^N\left( n_k -1/2 \right) }\prod _{k=1}^N p_{k,t}(n_k)\,, \end{aligned}$$

where the particle number conservation is taken into account by the Kronecker-delta for which we used a standard integral representation. The Bernoulli weights are

21 $$\begin{aligned} p_{k,t}(n_k) = n_k f_k(t)+(1-n_k)(1-f_k(t))\,, \end{aligned}$$

where $f_k\left( {t}\right) =\left( {1-t_k\left( {t}\right) }\right) /2$ with $t_k(t)=\mathrm {erf}\left( \Delta k/\sqrt{4{\widetilde{D}}\tilde{t}}\right)$ and $\Delta k = k-M-1/2$ is measured from the Fermi-level. Finally, the time-dependent normalization factor is the sum of all possible many-body probabilities:

22 $$\begin{aligned} \begin{aligned} \mathscr N_t\equiv \sum _{\left\{ n_k\right\} }P(\left\{ n_k\right\} )&=\int _{-\pi }^\pi \frac{\mathrm d\lambda }{2\pi }\prod _{k=1}^N\left[ e^{i \lambda /2}f_k(t)+e^{-i\lambda /2}(1-f_k(t))\right] =\int _{-\pi }^\pi \frac{\mathrm d\lambda }{2\pi }\prod _{k=1}^N\left[ \cos (\lambda /2)-i \sin (\lambda /2)t_k(t)\right] \\&=\int _{-\pi }^\pi \frac{\mathrm d\lambda }{2\pi }\prod _{\Delta k>0}\left[ \cos ^2(\lambda /2)+\sin ^2(\lambda /2)t_k(t)\right] \,. \end{aligned} \end{aligned}$$

Writing the above expression as the exponential of its logarithm, approximating the resulting sum by an integral and performing a saddle point approximation around $\lambda =0,$ we obtain for large enough values of $\langle w\rangle = {\widetilde{D}}{\tilde{t}} \gg 1$:

23 $$\begin{aligned} {\mathscr {N}}_t \approx \int _{-\pi }^\pi \frac{\mathrm d\lambda }{2\pi }\exp \left[ \int _0^\infty \mathrm dx\log \left( \cos ^2\lambda /2+t_x(t)\sin ^2\lambda / 2\right) \right] \approx \int _{-\pi }^\pi \frac{\mathrm d\lambda }{2\pi }\exp \left[ -\int _0^\infty \mathrm dx\lambda ^2/4(1-t_x(t))\right] =\left( 8\pi {\langle w\rangle }\right) ^{-1/4}\,. \end{aligned}$$

The probability of adiabaticity then reads

$$\begin{aligned} P_\text {ad}({\tilde{t}})&= \dfrac{1}{\mathscr {N}_t}\prod _{\Delta k<0} f_{k}(t) \prod _{\Delta k>0}\big (1- f_{k}(t)\big ) \\&\approx \dfrac{1}{\mathscr {N}_t} e^{2\sqrt{4{\langle w\rangle }}\int _0^\infty \! \mathrm{d}x \log [(1+\mathrm {erf}(x))/2]}={(8\pi {\widetilde{D}}\tilde{t})^{1/4}}\,{e^{-C\sqrt{{\widetilde{D}}\tilde{t}}}}={(8\pi {\langle w\rangle })^{1/4}}\,{e^{-C\sqrt{{\langle w\rangle }}}} \end{aligned}$$

with $$C\approx 1.35$$.

#### Variance of work

For $\langle w\rangle \gg 1$, we approximate the variance of the work by neglecting the fluctuations of the energy levels^56^ but incorporating the fluctuations of the occupation numbers. We thus measure the energies from the Fermi-level as $\varepsilon _k(t) \rightarrow \Delta k \,\delta \varepsilon$ with $\Delta k=k-M-1/2.$ For a given realization of ${{\mathscr {H}}}(t)$, this leads to the estimate

$$\begin{aligned} \delta w^2 (t) \approx \Big \langle \big ( \sum _{k = 1}^{N} \Delta k\, {\hat{n}}_{k,t}\big )^2\Big \rangle - \Big \langle \sum _{k = 1}^{N} \Delta k\, {\hat{n}}_{k,t} \Big \rangle ^2, \end{aligned}$$

where $\langle \dots \rangle$ denotes quantum average. Separating the diagonal terms, the RM average $\langle \delta w^2(t)\rangle _\mathrm{RM}$ can be written as

24 $$\begin{aligned} \langle \delta w^2(t)\rangle _{\mathrm{RM}} \approx \sum _{k }\Delta k^2\, \big \langle \big \langle \delta {\hat{n}}_{k,t}^2\big \rangle \big \rangle _{\mathrm{RM}} + \sum _{k \ne k'} \Delta k\,\Delta k' \big \langle \big \langle \delta {\hat{n}}_{k,t}\delta {\hat{n}}_{k',t} \big \rangle \big \rangle _{\mathrm{RM}} \,, \end{aligned}$$

where $\delta {\hat{n}}_{k,t} \equiv {\hat{n}}_{k,t} -\langle {\hat{n}}_{k,t}\rangle$ is the deviation of the occupation number from the mean value. As the ${\hat{n}}_{k,t}$ behave as binary random variables, the averages in the first term are given by $\langle \langle \delta {\hat{n}}_{k,t}^2\rangle \rangle _{\mathrm{RM}} = f_k(t) \big (1- f_k(t) \big )$. The correlators in this equation can be expressed in terms of the amplitudes $\alpha _k^m(t)$ as $\langle \delta {\hat{n}}_{k,t}\delta {\hat{n}}_{k',t}\rangle = - \big |\sum _{m=1}^{N/2} {\alpha _k^m (t)}^* \alpha _{k'}^m(t)\big |^2$. The negativity of this correction implies that the level occupations are *anticorrelated*, as follows from particle number conservation.

Neglecting this correction for the moment and replacing sums by integrals, we arrive at the estimate

$$\begin{aligned} \langle \delta w^2 (t)\rangle _{\mathrm{RM}} \approx \int _{-\infty }^{\infty } \mathrm{d}x\, x^2\, \frac{1 - \mathrm{erf}^2(x / \sqrt{4\widetilde{D}\tilde{t}})}{4} \sim \tilde{t}^{3/2}\,, \end{aligned}$$

yielding $\langle \delta w^2 (t)\rangle \sim \langle w\rangle ^{3/2}$. We thus recovered the observed behavior, however, the prefactor turns out to be incorrect. The correct prefactor^57^ can be obtained by a more careful mean field calculation that takes into account the occupation number correlations as well which obey that same scaling $\sim {\tilde{t}}^{3/2}.$

#### Distribution of work

The characteristic function of the distribution of work can be expressed as

25 $$\begin{aligned} \begin{aligned} G^\text {MF}_{{\tilde{t}}}\left( {u}\right)&=\left\langle e^{iu\sum _k\Delta k\,\delta \varepsilon \,n_k}\right\rangle _\mathrm {MF}e^{-i uE_\mathrm {GS}}=\sum _{\left\{ n_k\right\} }P_{\left\{ n_k\right\} }e^{i{\tilde{u}}\sum _k\Delta k(n_k-1/2)}\\&=\frac{1}{{\mathscr {N}}_t}\int _{-\pi }^\pi \frac{\mathrm d\lambda }{2\pi }\prod _{\Delta k} \left[ {e^{i\left( {\lambda +{\tilde{u}} \Delta k}\right) /2}f_k\left( {t}\right) +e^{-i\left( {\lambda +{\tilde{u}} \Delta k}\right) /2}f_{-k}\left( {t}\right) }\right] \\&=\frac{1}{{\mathscr {N}}_t}\int _{-\pi }^\pi \frac{\mathrm d\lambda }{2\pi }\prod _{\Delta k<0}\left[ {f_k(t)+e^{-i\left( {\lambda +{\tilde{u}} \Delta k}\right) }f_{-k}\left( {t}\right) }\right] \prod _{\Delta k>0}\left[ {f_{-k}(t)+e^{i\left( {\lambda +{\tilde{u}} \Delta k}\right) }f_k\left( {t}\right) }\right] \\&\approx \frac{1}{\widetilde{\mathscr N}_t}\int _{-\pi }^\pi \frac{\mathrm d\lambda }{2\pi } \exp \left[ {\int _0^\infty \mathrm dx\ln \left( {1+h^2_x\left( {u,t}\right) +2h_x\left( {u,t}\right) \cos \left( {\lambda }\right) }\right) }\right] , \end{aligned} \end{aligned}$$

where we introduced the scaled variable $\tilde{u}=u\,\delta \varepsilon$ and the notation $h_k(u,t)=\frac{f_k(t)}{{f_{-k}(t)}}e^{i{\tilde{u}} \Delta k}.$ Here $\langle \dots \rangle _\mathrm {MF}$ denotes averaging over the mean field many-body probabilities and $\widetilde{{\mathscr {N}}}_t$ a modified normalization constant. As numerics revealed, for large enough injected works ${\langle w\rangle }\gg 1$ neglecting particle number conservation does not introduce big errors provided we subtract the pure particle and pure hole excitations with respect to the ground state:

26 $$\begin{aligned} G^\text {MF}_{{\tilde{t}}}\left( {u}\right) \approx \frac{1}{\widetilde{\mathscr N}_t}\Bigg \{e^{2\int _0^\infty \mathrm dx\ln \left( {1+h_x\left( {t,u}\right) }\right) } -2\left[ {e^{\int _0^\infty \mathrm dx\ln \left( {1+h_x\left( {t,u}\right) }\right) }-1}\right] \Bigg \}, \end{aligned}$$

where the first term is the $\lambda =0$ saddle-point solution of the integral expression, while the second part substracts the contributions coming from the pure particle-hole excitations. Here the integrals can be approximated as

27 $$\begin{aligned} 2\int _0^\infty \mathrm dx\ln \left[ {1+h_x\left( {u,t}\right) }\right] \approx \frac{c^2_w}{\frac{c_w}{\sqrt{{\langle w\rangle }}}-iu} \end{aligned}$$

yielding

28 $$\begin{aligned} G^\text {MF}_{{\tilde{t}}}\left( {u}\right) \approx \frac{1}{\widetilde{\mathscr N}_t}\left\{ {e^{\frac{c^2_w}{\frac{c_w}{\sqrt{{\langle w\rangle }}}- iu}}-2\left[ {e^{\frac{c^2_w/2}{\frac{c_w}{\sqrt{{\langle w\rangle }}}-iu}}-1}\right] }\right\} \end{aligned}$$

with $c_w=\frac{3\sqrt{2\pi }}{5}$ chosen such that the characteristic function correctly reproduces the first two cumulants of work in the saddle point solution. Now, analogously to the Bosonization approach, both terms can be Fourier transformed exactly leading to the approximate analytic expression

29 $$\begin{aligned} P^\mathrm {\,MF} _{{\tilde{t}}}(w) \approx e^{-c_w\sqrt{{\langle w\rangle }}}\left[ {e^{-\frac{c_w}{\sqrt{{\langle w\rangle }}}w}\frac{c_w}{\sqrt{w}} \left( {I_1(2c_w\sqrt{w})-\sqrt{2}I_1\bigl ( 2c_w\sqrt{ w/2}\bigr )}\right) +\delta \left( {w}\right) }\right] \,. \end{aligned}$$

### Energy space diffusion

**Figure 4:**
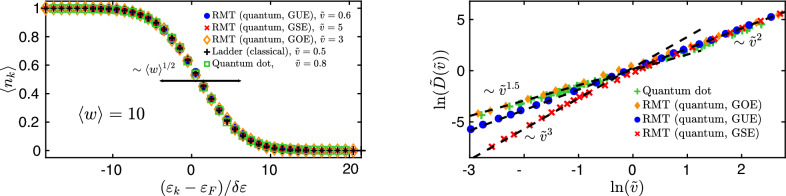
Energy space diffusion. *Left:* Average occupations of instantaneous single particle eigenstates for the three RMT ensembles (GOE, GUE, GSE) and the quantum dot model compared to the classically obtained results within the ladder model. All the five curves collapse onto a single universal, diffusively broadening profile given by $\left[ {1-\mathrm {erf}\left( {\Delta k/\sqrt{4{\langle w\rangle }}}\right) }\right] /2$. *Right:* Velocity dependence of the diffusion constant. For slow quenches it has an anomalous power-law behavior, ${\widetilde{D}}({\tilde{v}}\lesssim 1)\sim {\tilde{v}}^{\beta /2+1}$, while for fast quenches it grows quadratically and with the same prefactor for the RMT ensembles. The quantum dot displays a similar behavior as the GOE ensemble in the two limiting cases, with a slightly different prefactor. In the left panel, we used $N=40$ energy levels for the GOE and GSE, $N=28$ for the GUE ensemble, and $N=56$ for the ladder model simulations, and half filling in all cases. For the quantum dot model, the same parameters were used as in Fig. 3. We used various values of RMT and quantum dot parameters to obtain the results shown in the right panel.

In this section we demonstrate that the energy level occupations exhibit a diffusive profile, meaning that particle-hole excitations happen dominantly in a window growing as $\sim {\langle w\rangle }^{1/2}$, for all the random matrix ensembles as well as for the “ladder model” and the disordered quantum dot. The left panel of Fig. 4 shows that for large enough average work the mean level occupation for of all three RMT ensembles (GOE, GUE, GSE) follows a single universal curve identical to those of the quantum dot model up to high precision and it is also perfectly described by the ladder model. Numerical calculations were made for $\sim 5\times 10^3$ disorder realizations both for random matrix theory and the disordered quantum dot, for $N=40,28,40$ for the three ensembles, respectively and for parameters $L=38,\sigma =1.75J,\alpha =75J$ and with 427 particles in the case of the quantum dot.

The right panel of Fig. 4 shows the velocity dependence of the diffusion constant, ${\widetilde{D}}_\beta ({\tilde{v}})$ for the three ensembles and the quantum dot model. We averaged over $\sim 5\times 10^3$ simulations, yielding smooth enough time-evolutions of average work to extract the diffusion constants. Parameters were chosen such that we avoid finite size effects and be in the diffusion regime. The rate of energy absorbed by the system exhibits an anomalous frequency dependence for slow quenches, ${\widetilde{D}}_\beta ({\tilde{v}}\lesssim 1)\sim {\tilde{v}}^{\beta /2+1}$, while for fast processes becomes independent of the underlying symmetry class and grows quadratically, as it should in the case of a metal, ${\widetilde{D}}_\beta ({\tilde{v}}\gg 1)\sim {\tilde{v}}^2$. The diffusion constant for the quantum dot shows the same power-law behavior as the GOE ensemble, albeit with a slightly smaller prefactor.

**Figure 5:**
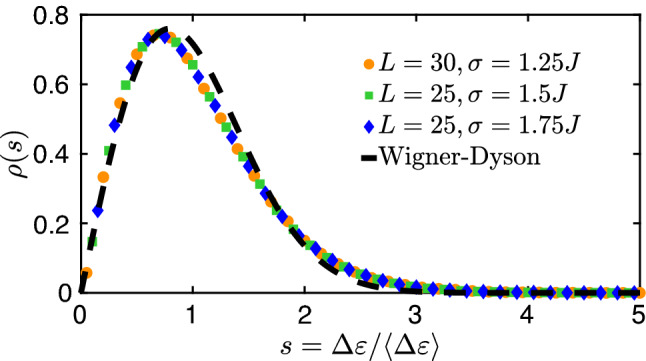
Distribution of the distance between neighboring levels in the middle of the spectrum, $\Delta \varepsilon \equiv \varepsilon _{L^2/2+1}-\varepsilon _{L^2/2}$, normalized to unit mean, for the quantum dot at three different set of parameters, ($L=30,\sigma =1.25J$), ($L=25,\sigma =1.5J$), ($L=25,\sigma =1.75J$) for the orange circles, green squares, and blue diamonds, respectively. The potential parameters were fixed to $\alpha =70J$ and $\lambda =0$ (symmetric case) for all three curves (there are no relevant differences for small deformations by a finite $\lambda$). The dashed line indicates the well-known Wigner–Dyson result, $\rho (s)\approx \frac{\pi }{2}s\,e^{-\frac{\pi }{4}s^2}$, obtained by Wigner’s surmise describing the GOE case. For the numerical calculations we averaged over $\sim 5\times 10^4$ disorder realizations which proved to yield smooth enough curves.

Finally, we compare the level spacing distribution of the GOE ensemble and the disordered quantum dot. As shown in Fig. 5, the distribution of the distance of neighboring levels are well described by the analytical RMT result given by the Wigner surmise. Similar observations hold for the statistics of the the Landau–Zener parameters at the avoided level crossings in comparison with the RMT results of Ref.^37^.

## Data Availability

The datasets used and/or analysed during the current study available from the corresponding author on reasonable request.
